# Chemical Pattern Recognition and Color–Chromaticity Correlation Analysis for Quality Control of Stir-Fried Perillae Fructus

**DOI:** 10.3390/molecules31111907

**Published:** 2026-06-02

**Authors:** Liangying Li, Xiaobin Deng, Pengbo Wang, Nina Zeng, Jing Hu

**Affiliations:** 1 School of Chinese Materia Medica, Tianjin University of Traditional Chinese Medicine, Tianjin 301617, China; liliangying2023@163.com (L.L.); dengxiaobin10@163.com (X.D.); 18202656176@163.com (P.W.); 13978348670@163.com (N.Z.); 2 Tianjin Key Laboratory of Therapeutic Substance of Traditional Chinese Medicine, Tianjin 301617, China

**Keywords:** Perilla Fructus, fingerprint, quality evaluation, chemical pattern recognition, correlation analysis

## Abstract

**Objective**: Perillae Fructus (PF) (*Perilla frutescens* (L.) Britt.) and stir-fried Perillae Fructus (SFPF) are commonly used clinically for the treatment of cough and asthma, yet their quality control methods have not been fully established. **Method**: The best processing techniques of PF were optimized by one-variable-at-a-time (OVAT) analysis and Box–Behnken design (BBD); fingerprint combined with chemical pattern recognition techniques was employed to establish chromatographic fingerprints of PF and SFPF from different regions. Differential compounds were screened and the reliability of the established method was verified through quantitative analysis of multi-components; image processing technology was applied to determine chromaticity values and perform cluster heatmap analysis. The composition–color correlation of PF and SFPF was investigated. **Result**: Four characteristic components were identified through 36 batches of PF and SFPF, with rosmarinic acid, 5-hydroxymethylfurfural, caffeic acid and luteolin serving as discriminant markers differentiating PF and SFPF. The contents of seven components and the corresponding chromaticity parameters (L*, a*, b*) were determined to generate a visualized heatmap. Rosmarinic acid and caffeic acid showed positive correlations with L*, whereas a negative correlation was shown with b* and 5-hydroxymethylfurfural. **Conclusions**: This study provides a theoretical basis for judgment of processing endpoints and the rapid online quality monitoring of SFPF.

## 1. Introduction

*Perilla frutescens* (L.) Britt. (also called Zisu in China), an annual herbaceous plant, is widely distributed worldwide, including China, Japan, Korea, Vietnam and other regions in Asia. Perillae Fructus (PF) is the first plant recognized as homologous to food and medicine by the Ministry of Health [1]. Stir-fried Perillae Fructus (SFPF) is the most extensively applied processed form in clinical practice. Classical text records in the MingYi BieLu first mention PF, highlighting stir-frying as its primary traditional processing technique [2]. In the processing theory of traditional Chinese medicine (TCM), stir-frying moderates the pungent and drying properties of PF and also enhances the effects of relieving coughs and asthma. which has also been verified by modern pharmacological studies [1,3,4]. Nevertheless, the processing methods of SFPF largely follow traditional practices. Processing parameters vary significantly across different production regions and manufacturers. The endpoint of processing is often determined by sensory evaluation rather than quantitative indicators. Due to inconsistent processing methods and unstable heat control, SFPF from different manufacturers and batches exhibit significant variations in appearance, color, aroma, and chemical composition. This results in poor quality uniformity and instability, directly affecting the safety, efficacy, and quality control of SFPF in clinical applications. Therefore, establishing a scientific, quantifiable, and reproducible processing method is of great significance for ensuring the stable quality and safety of SFPF. Furthermore, current research on processing procedures mainly concentrates on single compounds including rosmarinic acid and luteolin [5]; studies on general components such as total polysaccharides (TPs), total phenolic acids (TPAs) and total flavonoids (TFs) remain limited. Perilla Fructus oil (PFO) contains approximately 60% *α*-linolenic acid, representing one of the highest levels among vegetable oils, and has attracted extensive attention owing to its diverse pharmacological activities [6,7].Therefore, these indicators were selected to further clarify the processing techniques of SFPF, which is crucial for quality assurance and standardization efforts.

In the *Chinese Pharmacopeia*, rosmarinic acid is only used as a quality marker. Extensive research indicates that rosmarinic acid possesses a wide range of potential biological activities [8,9,10]. However, the therapeutic efficacy of TCM typically relies on the synergistic action of multiple components. Evaluating its overall effect based on a single compound fails to comprehensively reflect its clinical efficacy. Furthermore, numerous plants are abundantly distributed in rosmarinic acid, e.g., *Thymus vulgaris*, *Pulmonaria officinalis* and *Salvia officinalis*, lacking specificity to SFPF [11]. The TCM fingerprinting, which provides abundant chemical composition information, is an effective tool for the holistic quality evaluation of TCM [12,13]. Chemometric methods such as principal component analysis (PCA) and orthogonal partial least squares discriminant analysis (OPLS-DA) can efficiently explore characteristic differences in complex systems [14,15,16,17].

Traditional quality control of TCM relies on empirical identification, which mainly involves observing color with eyes, feeling texture by touch, smelling odor, and tasting flavor. However, such conventional identification approaches are highly dependent on personal subjective experience and lack objective and quantitative criteria [18]. Therefore, modern bionic technologies have introduced electronic eye techniques for the objective analysis of TCM quality. Yang et al. employed electronic eye technology to obtain color parameters combined with various machine learning algorithms, and established a classification model for the geographical origin of *Atractylodis Macrocephalae Rhizoma* [19]. Le et al. applied electronic eye to establish fingerprints for raw *Acori Tatarinowii Rhizoma* and bran-fried *Acori Tatarinowii Rhizoma*. The results showed that bran-fried *Acori Tatarinowii Rhizoma* exhibited darkening of the samples, an increase in red dominance, and a slight increase in yellow dominance, providing support for the quality control of TCM [20]. Nevertheless, electronic eye technology is still limited to some extent by application scenarios, equipment cost, and other factors, which restrict its further development.

In summary, this study employed the Box–Behnken design (BBD) combined with response surface methodology (RSM) to optimize the processing parameters and obtain stable experimental samples of PF and SFPF. High-performance liquid chromatography (HPLC) fingerprinting was established to reveal the differential chemical constituents between PF and SFPF. A new algorithm-based method was proposed to extract color values (L*, a*, b*, ΔE*) from images of PF and SFPF. The contents of seven chemical components were determined and further correlated with the color values, so as to clarify the key chemical constituents responsible for color variation in SFPF, and to improve the comprehensive quality control method for SFPF.

## 2. Results

### 2.1. One-Variable-at-a-Time (OVAT) Analysis

**Figure 1:**
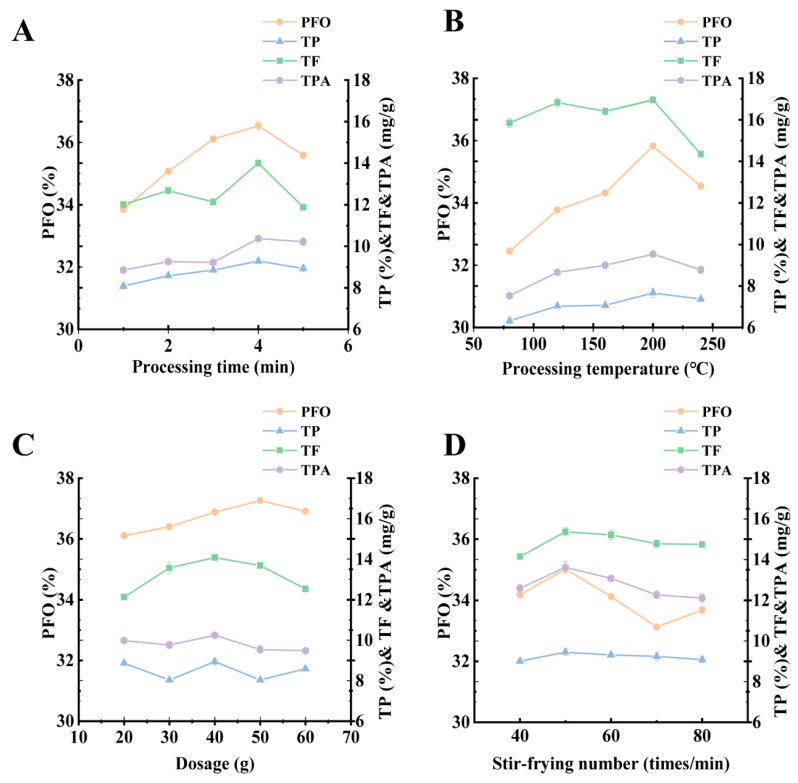
Results of OVAT analysis. (**A**) Effects of different processing times on the PFO, TF, TPA and TP of SFPF, (**B**) effects of different processing temperatures on the PFO, TF, TPA and TP of SFPF, (**C**) effects of different dosages on the PFO, TF, TPA and TP of SFPF, (**D**) effects of different stir-frying numbers on the PFO, TF, TPA and TP of SFPF.

Figure 1 describes the comprehensive study of yield trends for four components (PFO, TP, TF and TPA). Factors discussed include processing time, processing temperature, dosage, and stir-frying number. The four contents gradually increased before decreasing. For subsequent experiments, we selected three levels (−1, 0, 1) as the experimental basis, with detailed conditions as follows: processing temperature: 160 °C (−1), 200 °C (0), and 240 °C (1); processing time: 3 min (−1), 4 min (0), and 5 min (1); dosage: 30 g (−1), 40 g (0), and 50 g (1); stir-frying number: 40 times/min (−1), 50 times/min (0), and 60 times/min (1).

### 2.2. Response Surface Methodology Experiment

**Figure 2:**
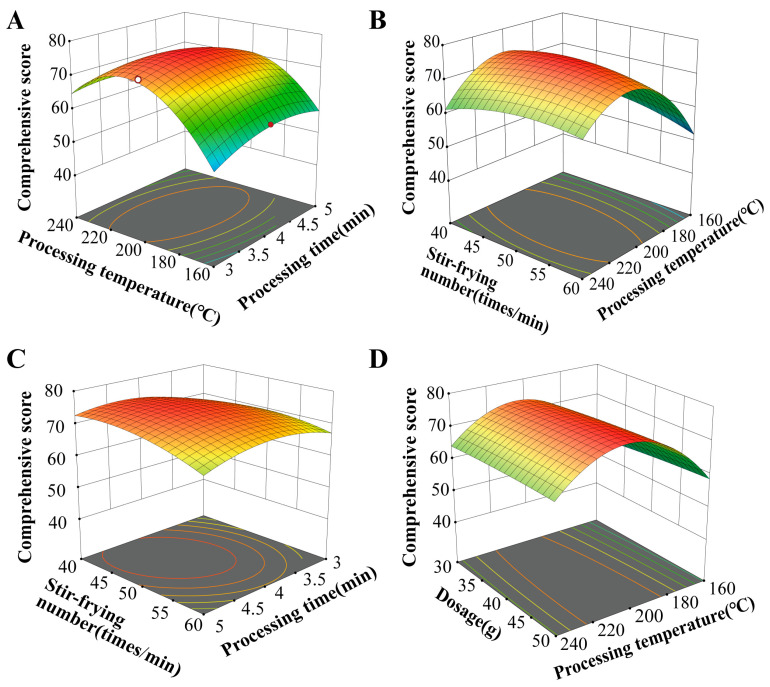
Result of RSM. (**A**) 3D surface map of processing time and processing temperature, (**B**) 3D surface map of processing temperature and stir-frying number, (**C**) 3D surface map of processing time and stir-frying number, (**D**) 3D surface map of temperature and dosage.

Based on the OVAT analysis, four variables (PFO, TP, TPA, and TF) were selected for their impact on the comprehensive score. Subsequently, the contents of the four compounds were calculated and input into Design Expert 8.0 software. Twenty-nine experimental groups were designed (Table S2), yielding the following polynomial equations: Y = 77.51 + 2.22A + 3.65B + 0.1717C − 1.01D − 2.49AB − 2.89AC − 1.63AD − 0.455BC + 2.37BD − 0.0350CD − 3.78A^2^ − 13.36B^2^ − 0.3792C^2^ − 2.72D^2^ (A: processing temperature, B: processing time, C: stir-frying frequency, D: dosage). Analysis of variance (ANOVA) results (Table S3) showed significant differences in SFPF models (*p* < 0.0001) and the models had good fits (R^2^ 0.9685), and most of the response variability was explained. Ultimately, based on the experimental results (Figure 2, Tables S2 and S3), the optimized process parameters were determined as follows: processing temperature of 200 °C, processing time of 4.5 min, stir-frying number of 45 times/min, and dosage of 30 g. Furthermore, based on the RSM data analysis, experimental validation (*n* = 3, Table S4) gave a comprehensive score of 79.09 (RSD < 3%). No significant difference was observed between experimental and predicted values (*p* > 0.05). The predicted results closely matched the experimental outcomes obtained under optimal extraction conditions, thereby validating the excellent applicability of the BBD-RSM model.

This study employs the RSM for the first time to systematically optimize the processing techniques of SFPF. Based on the experimental results, the following conclusions can be drawn: higher temperatures and longer heating times induce significant chemical transformations; specifically, thermal effects promote degradation reactions of macromolecular substances while accelerating the oxidation rate of easily oxidized components such as fats and polyunsaturated fatty acids; the Maillard reaction facilitates interactions between amino acids and reducing sugars, leading to the formation of a series of flavor compounds and browned products [21,22,23].

### 2.3. HPLC Fingerprint Analysis of PF and SFPF

#### 2.3.1. Establishing the Fingerprint of PF and SFPF

**Figure 3:**
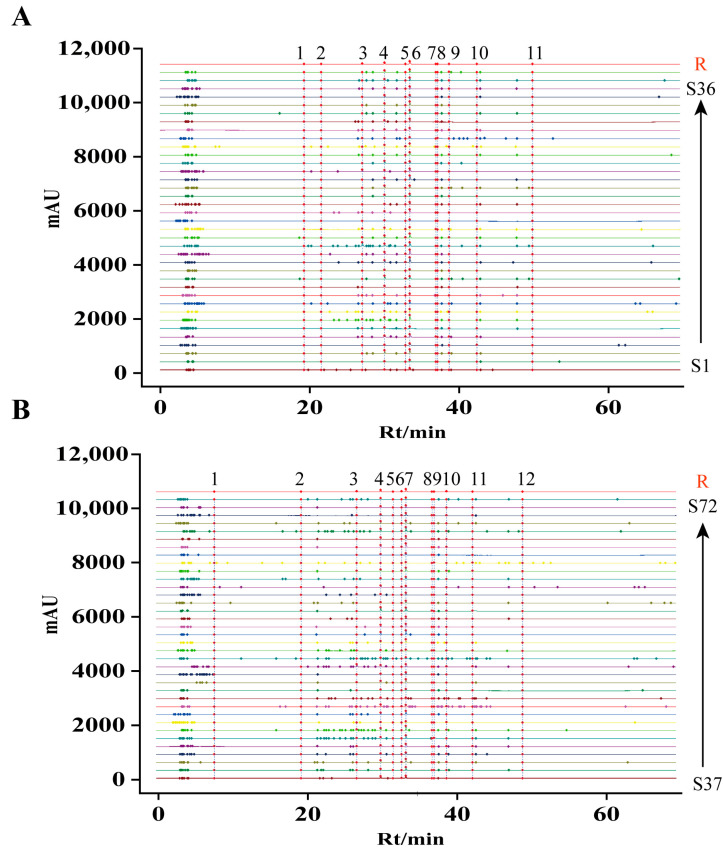
HPLC fingerprint of PF (**A**) and SFPF (**B**).

**Figure 4:**
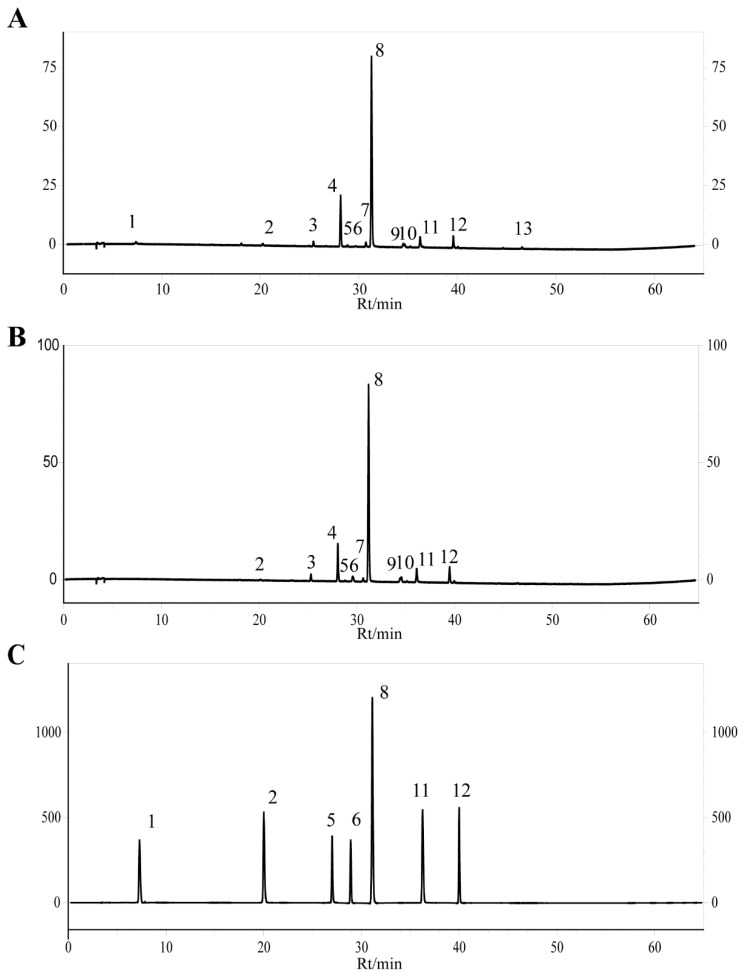
HPLC chromatogram of PF, SFPF and reference solution. (**A**) PF, (**B**) SFPF, (**C**) reference solution; peak 1: 5-HMF; peak 2: caffeic acid; peak 5: luteolin-7-O-glucoside; peak 6: apigenin-7-O-glucoside; peak 8: rosmarinic acid; peak 11: luteolin; peak 12: apigenin.

Data from 36 batches of PF (S1–S36) and SFPF (S37–S72) samples were imported in “AIA” format into the Chinese Materia Medica chromatographic fingerprint similarity evaluation system (2012 version), using S5 as the reference peak, with a time window width of 0.2 min. Multi-point correction was performed via full-spectrum peak matching, and fingerprints of PF and SFPF were constructed using the median method. Based on the determination of the chemical components contained in the HPLC analysis, fingerprint chromatograms were collected for 36 batches of PF and SFPF. Subsequently, the similarity analysis was performed. A total of 11 common peaks were detected in 36 batches of PF (Figure 3A), with similarity values ranging from 0.913 to 0.997 (Table S7). For 36 batches of SFPF, 12 common peaks were identified (Figure 3B), and their similarities varied between 0.914 and 0.997 (Table S8). The inconsistency in the similarity ranges between PF and SFPF indicates that processing could modify the chemical composition of PF. Eleven common peaks were shared by PF and SFPF, among which seven components were identified as 5-hydroxymethylfurfural (5-HMF), caffeic acid, luteolin-7-O-glucoside, apigenin-7-O-glucoside, rosmarinic acid, luteolin, and apigenin (Figure 4). Methodological validation of the common peaks in the fingerprints of PF and SFPF is presented in Table S4.

#### 2.3.2. Chemical Pattern Recognition Analysis

**Figure 5:**
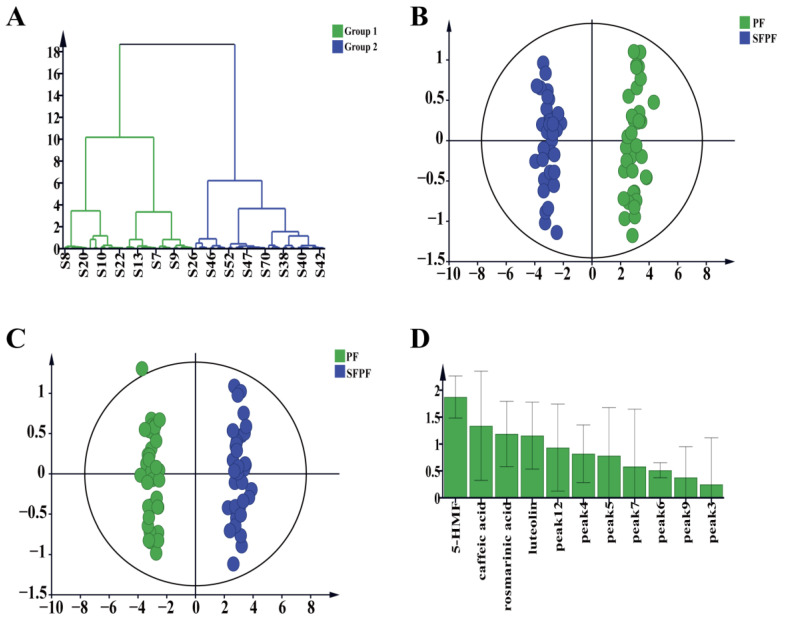
Chemical pattern recognition of chemicals. (**A**) HCA score scatter plot of PF and SFPF, (**B**) PCA score scatter plot of PF and SFPF, (**C**) OPLS-DA score scatter plot of PF and SFPF, (**D**) VIP plot of PF and SFPF.

Hierarchical cluster analysis (HCA) was performed using Simca 14.0 software based on the peak areas of 11 common peaks in PF and SFPF (missing values were recorded as 0). The results are shown in Figure 5A. PF and SFPF were mainly clustered into two categories: S1–S36 were clustered into one class, and S37–S72 were clustered into another class, indicating differences in chemical compositions between PF and SFPF. PCA of 36 batches of PF and SFPF showed that the 72 samples from 12 provinces were clearly distinguished (Figure 5B). Meanwhile, the PCA model yielded an R^2^ value of 0.865 and a Q^2^ value of 0.614, demonstrating good model fit. These results were in agreement with the conclusions of cluster analysis, further confirming the existence of significant variety differences among samples. The results of HCA and PCA could corroborate each other. For OPLS-DA analysis, the score scatter plot of the OPLS-DA model (Figure 5D) showed that PF and SFPF were essentially separated.

The core criterion for screening quality markers is the variable importance projection (VIP) value. The effect strength of chemical biomarkers in inducing intergroup differences increases with rising VIP values. The results of Figure 5D indicate that four peaks with VIP > 1 can identify significant intergroup differences in quality markers. After comparison with reference standards, these were specifically identified as rosmarinic acid (peak 8), 5-hydroxymethylfurfural (peak 1), caffeic acid (peak 2) and luteolin (peak 11), as shown in Table 1. These results suggested that the HPLC fingerprint can comprehensively characterize the overall quality of TCM under an integral analytical mode, and provide technical support for the establishment of a comprehensive quality evaluation system based on multi-index components, which is of important application value for the quality control of TCM and the discovery of differential compounds.

#### 2.3.3. Result of Methodological Validation

As shown in Table 2, the seven compounds exhibited good linear relationships within certain concentration ranges (R^2^ > 0.9970). The instrument precision was high with RSD values ranging from 0.29% to 0.45%. The sample solutions were stable within 10 h. The RSD values of the repeatability test were between 0.85% and 1.97%, and the recoveries of the spiked samples ranged from 98.08% to 102.05%. These results demonstrated that the established method was reliable and suitable for quantitative analysis.

#### 2.3.4. Analysis of Chemical Composition Content

**Figure 6:**
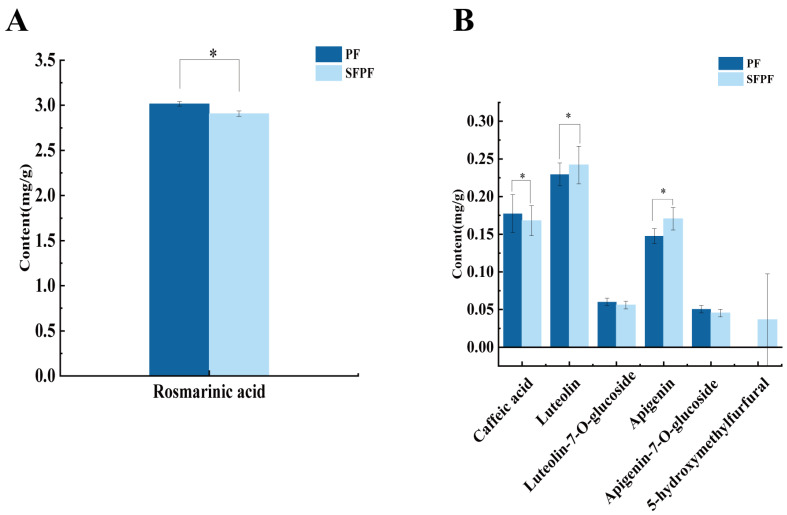
Content determination of differential compounds between PF and SFPF. (**A**) Changes in rosmarinic acid content. (**B**). Changes in caffic acid, luteolin, luteolin-7-O-glucoside, apigenin, apigenin 7-O-glucoside and 5-hydroxymethylfurfural content. ($\overset{-}{x}$ ± *s*, *n* = 3, * represent *p* < 0.05).

Based on the above research, the established method was applied to determine the contents of rosmarinic acid, caffeic acid, luteolin, apigenin, luteolin-7-O-glucoside, apigenin-7-O-glucoside, and 5-HMF in 36 batches of PF and SFPF. The results are presented in Figure 6 and Tables S7 and S8. As shown in the figures, compared with PF, the contents of rosmarinic acid and caffeic acid in SFPF were significantly decreased (*p* < 0.05), whereas the contents of flavonoids such as luteolin and apigenin were significantly increased (*p* < 0.05). In addition, 5-hydroxymethylfurfural was newly detected after processing. These compounds could serve as differential markers to distinguish between PF and SFPF.

### 2.4. Analysis of the Relationship Between Color and Composition of PF and SFPF

#### 2.4.1. Color Measurement Analysis of PF and SFPF

**Figure 7:**
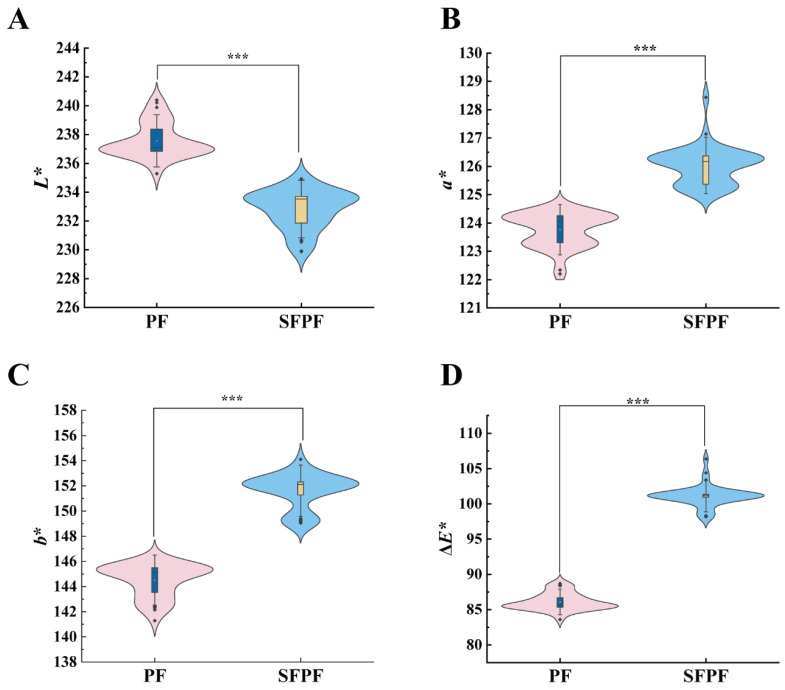
Distribution range of sample color difference values L* (**A**), a* (**B**), b* (**C**) and ΔE* (**D**) (*** represent *p* < 0.001 extremely significant).

Color parameters were collected for 36 batches of PF and SFPF using an algorithm. Each batch was tested in triplicate, and the mean values were subjected to statistical analysis, as shown in Figure 7. The results indicate that there is some variation in color among different batches of PF and SFPF. Compared with the PF, the L* of SFPF decreased significantly (*p* < 0.001), indicating that brightness decreased and color deepened after processing. The a* showed an upward trend (*p* < 0.001), indicating a slight intensification of the red hue, while the b* increased extremely significantly (*p* < 0.001), indicating a marked deepening of the yellow hue. The ΔE* color difference values for SFPF powder before and after processing across 36 batches ranged from 15.68 to 3.0, all exceeding 3.0, indicating that the color differences before and after processing reached a level clearly distinguishable to the eye. In summary, processing significantly altered the color of the samples, primarily manifested as reduced brightness and a marked enhancement of the yellow hue.

#### 2.4.2. Correlation Analysis of the Color and Composition of SFPF

**Figure 8:**
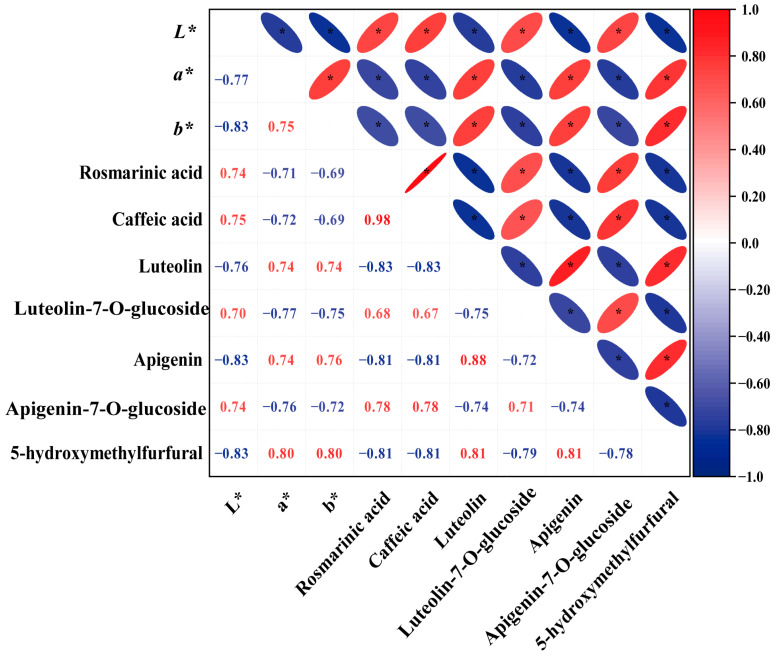
Correlation analysis between color difference values and active ingredient contents of SFPF (* represent *p* < 0.05).

A correlation analysis was conducted between the apparent color of SFPF and the contents of seven components, and the results are shown in Figure 8. It can be seen that the L* is positively correlated with rosmarinic acid and caffeic acid (*p* < 0.05) and negatively correlated with 5-HMF; luteolin and apigenin are positively correlated with the b* (*p* < 0.05). The results of the correlation analysis indicate that as the content of rosmarinic acid and caffeic acid in SFPF increases, the L* (lightness value) of the sample increases; conversely, as the b* (yellow–blue hue) of SFPF increases, the content of 5-HMF, luteolin, and apigenin increases.

#### 2.4.3. Regression Analysis of the Color and Composition of SFPF

Using the color difference indices L*, a*, and b* as independent variables, and the content of rosmarinic acid, caffeic acid, luteolin, apigenin, luteolin-7-O-glucoside, apigenin-7-O-glucoside, and 5-HMF as the dependent variables, we performed regression analysis using SPSS 27.0 software. The R^2^ values for the L*, a*, and b* models were 0.03, 0.03, and 0.03, respectively; for rosmarinic acid, caffeic acid, luteolin, apigenin, luteolin-7-O-glucoside, apigenin-7-O-glucoside and 5-hydroxymethylfurfural they were 0.032, 0.014, 0.198, 0.023, 0.177, 0.246, and 0.024, indicating that 3.2%, 1.4%, 19.8%, 2.3%, 17.7%, 24.6%, and 2.4% of the active components in SFPF—namely rosmarinic acid, caffeic acid, luteolin, and 5-hydroxymethylfurfural—are influenced by the color difference indices L*, a*, and b*. A regression analysis was performed on the content of the five active components—rosmarinic acid, caffeic acid, luteolin, apigenin, and 5-HMF—which are statistically significant (R^2^ > 10%), in relation to the color difference indices. A regression analysis of the color difference values and active ingredient content data using SPSS 27.0 yields the following regression equation: Y _rosmarinic acid_ = −74.617 + 0.945L* − 0.420a* − 0.594b*, Y _caffeic acid_ = −8.155 + 0.060L* − 0.001a* − 0.038b*, Y _luteolin_ = 8.945 − 0.107L* + 0.021a* + 0.090b*, Y _apigenin_ = 5.605 − 0.030L* − 0.010a* + 0.017b*, Y_5-HMF_ = 4.918 − 0.023L* − 0.011a* + 0.012b*. Although the regression models exhibited relatively low R^2^ values and thus lacked high precision for accurate quantitative prediction, color difference parameters still presented clear correlation trends with the contents of the five active components. The established equations can reflect the changing tendency of component contents and provide a preliminary intuitive reference for rapid qualitative screening and holistic quality assessment of SFPF based on chromatic characteristics, rather than precise quantitative determination.

## 3. Materials and Methods

### 3.1. Materials and Reagents

Thirty-six batches of PF were identified as *Perilla frutescens* (L.) Britt Fructus by professor Tiangxiang Li at the Tianjin University of Traditional Chinese Medicine. Detailed information for the thirty-six batches PF is presented in Table S1.

Rosmarinic acid (NO. PS1278-0020, 98.5%), caffeic acid (NO. PU1191-0025, 98%), luteolin (NO. PU0033-0025, 99%), luteolin-7-O-glucoside (NO. PU0264-0025, 98.5%), apigenin (NO. PU0158-0025, 99%), apigenin 7-O-glucoside (NO. PS2006-0020, 98%), and 5-hydroxymethylfurfural (NO. PS1517-0100, 95%, 5-HMF) were acquired from Push Biotechnology Co., Ltd. (Chengdu, China). Gallic acid (NO. MFCDG666651, 95%) and anhydrous D-glucose (NO. MFCD810588, 98%) were purchased from Macklin Biochemical Technology Co., Ltd. (Shanghai, China). Rutin (NO. B25342, 98%) was purchased from Sourceleaf Biotechnology Co., Ltd. (Shanghai, China).

HPLC grade methanol, acetonitrile, and formic acid were obtained from Fisher Scientific (Pittsburgh, PA, USA). Ultrapure water was prepared by a Milli-Q system (Millipore, Molsheim, France). Other chemicals and reagents were of analytical grade.

### 3.2. Optimization of Processing Procedures of SFPF

#### 3.2.1. OVAT Analysis

The optimum processing techniques of SFPF are determined by a series of experiments, including the dosages (20 g, 30 g, 40 g, 50 g, 60 g), the processing temperature (80 °C, 120 °C, 160 °C, 200 °C, and 240 °C), the processing time (1 min, 2 min, 3 min, 4 min, and 5 min), and the stir-frying numbers (40 times/min, 50 times/min, 60 times/min, 70 times/min, 80 times/min). The OVAT experiment uses these four factors for screening design. When evaluating the effect of a single variable on content, the remaining variables are kept constant. Therefore, only one variable is changed at a time to determine the optimal conditions.

#### 3.2.2. Response Surface Methodology (RSM) Experimental Design

Box–Behnken experimental design (BBD) is extensively applied in analytical chemistry and process optimization for pharmaceutical preparations, offering the advantage of identifying optimal process conditions with relatively few experiments [23]. Through OVAT analysis, four key parameters requiring further optimization were preliminarily identified. Within the BBD framework, a four-factor, three-level RSM was employed for experimentation. All experimental design, regression analysis, and data evaluation were performed using Design-Expert software (v13.0). The specific experimental plan and results are detailed in Table S2. ANOVA was conducted on the experimental data, and the results were fitted into a complete quadratic polynomial model (Table S3). As referenced in the literature, the CRITIC method was applied to determine the weight of evaluation indicators PFO, TP, TF and TPA [24]. The comprehensive score was calculated using the following formula:

$$Y=\left(0.458\;\times \;\frac{A}{\mathrm{Amax}}\;+\;0.299\;\times \;\frac{B}{\mathrm{Bmax}}\;+\;0.139\;\times \;\frac{C}{\mathrm{Cmax}}\;+\;0.103\;\times \;\frac{D}{\mathrm{Dmax}}\right)\times \;100$$

Y is the comprehensive score, A is the TPAC standard value, B is the TFC standard value, C is the TPC standard value, and D is the PFO standard value.

#### 3.2.3. Chemical Extraction

PF and SFPF were powdered and screened through 24 mesh sieves. A certain amount of the sample was weighed, and petroleum ether was added at a solid-to-liquid ratio of 10:1. After thorough mixing and standing for 1 h, ultrasonic extraction was carried out at 30 °C for 2h (500W, 40kHz). Filter the extract and the defatted powder will be collected.

TP extraction: Defatted powder (1.0 g) was weighed accurately and thoroughly mixed with 25 mL ultrapure water; ultrasonic extraction was carried out at 40 °C for 50 min, the mixture was centrifuged (5000 rpm/min for 5 min), and the supernatant was collected for contents determination of total polysaccharide.

TF and TPA extraction: Defatted powder (0.5 g) was weighed accurately and thoroughly mixed with 75% (*v*/*v*) methanol (10 mL), then extracted by ultrasonic (500 W, 40 kHz) extraction for 30 min. The mixture was centrifuged (5000 rpm/min for 5 min), and the supernatant was collected for contents determination of total flavonoids and total phenolic acids.

#### 3.2.4. Determination of PFO

The extraction and determination method of PFO was slightly modified according to the reported literature [25]. Briefly, PF was pulverized and extracted with petroleum ether at a solid–liquid ratio of 10:1. After removal of the solvent, the resulting liquid was defined as Perilla Fructus oil (PFO). The weight of the evaporating dish was measured before and after evaporation, and the yield was calculated according to the following formula.

$$Y\left(\%\right)=\left[\mathrm{Mass}\;\mathrm{of}\;\mathrm{oil}\;(g\right)/\mathrm{Mass}\;\mathrm{of}\;\mathrm{sample}\;(g)]\times \;100\%$$

where Y is the yield of PFO (%), mass of oil refers to the weight of the evaporating dish before solvent removal minus the weight of the evaporating dish after solvent removal (g), and mass of SFPF is the weight of the sample taken (g).

#### 3.2.5. Determination of TP

The extraction and determination method of TP was modified slightly according to Duan et al. [26]. Quantitative analysis of TP was performed by using D-glucose as the standard to plot a calibration curve (Y = 15.09X + 0.1057, linear range: 0.0025–0.0125 mg/mL, R^2^ = 0.9996). TP was calculated using the following formula:

Y (%) =C × N × V/M

where Y is the yield of TP from SFPF (%); C denotes the polysaccharide concentration (μg/mL) calculated; N is the dilution factor; V is the volume of the sample solution (mL); and M is the mass of SFPF (g).

#### 3.2.6. Determination of TF

The extraction and determination method of TF was modified slightly according to a previous report [27]. For quantitative analysis of TF, a standard curve was established using rutin as the standard (Y = 10.163 X + 0.0621, linear range: 0.01–0.06 mg/mL, R^2^ = 0.9994). The TF was calculated according to the following formula:

$$Y(\mathrm{mg}/g)\;=C_{1}\;\times \;N_{1}\;\times \;V_{1}/M_{1}$$

where Y is the yield of TF from SFPF (mg/g); C_1_ is the flavonoid concentration (mg/mL); N_1_ is the dilution factor; V_1_ is the volume of the sample solution (mL); and M_1_ is the mass of SFPF (g).

#### 3.2.7. Determination of TPA

The extraction and determination method of TPA was modified slightly according to a previous report [28]. For quantitative analysis of TPA, a standard curve was established using gallic acid as the standard (Y = 87.486 X + 0.1133, linear range: 0.02~0.12 mg/mL, R^2^ = 0.9991). The TPA extraction rate was calculated according to the following formula:

$$Y(\mathrm{mg}/g)\;=C_{2}\;\times \;N_{2}\;\times \;V_{2}/M_{2}$$

where Y is the yield of TPA from SFPF (mg/g); C_2_ is the concentration of TPA in the test solution (mg/mL); N_2_ is the dilution factor; V_2_ is the volume of the sample solution (mL); and M_2_ is the mass of SFPF (g).

### 3.3. HPLC Fingerprint Analysis of PF and SFPF

#### 3.3.1. Preparation of HPLC Solution

Preparartion of SFPF: Take 30 g of PF respectively and remove impurities. The samples were processed following the optimal proceessing conditions described in Section 2.2.

Test solution preparation: Accurately weigh 0.25 g sample of PF and SFPF powder; 10 mL of 80% (*v*/*v*) methanol solution was added and sonicated at 40 °C for 30 min. Then, the mixture was cooled to room temperature and centrifuged at 1000 r/min for 10 min. The supernatant was filtered through a 0.22 μm microporous membrane and used for HPLC analysis [29]. A total of 72 samples of PF (S1–S36) and SFPF (S37–S72) was prepared for the test solution.

Mixed reference solution preparation: Appropriate amounts of rosmarinic acid, caffeic acid, luteolin, luteolin-7-O-glucoside, apigenin, apigenin-7-O-glucoside and 5-hydroxymethylfurfural reference materials were accurately measured, dissolved and combined with methanol in 10 mL volumetric flasks at concentrations of 0.3 mg/mL, 0.03 mg/mL, 0.5 mg/mL, 0.5 mg/mL, 0.012 mg/mL, 0.012 mg/mL, and 0.012 mg/mL. The mixture was shaken well and filtered through a 0.22 µm microporous membrane. The filtrate was then taken for HPLC analysis.

#### 3.3.2. HPLC Conditions

An Alltech 1500 series II system HPLC-UV system (Alltech Associates, Inc., Chicago, IL, USA) equipped with a Hypersil GOLD-C18 chromatographic column (4.6 × 250 mm, 5 μm) was utilized to analyze PF and SFPF samples. The mobile phase comprised 0.01% formic acid in water (solvent A) and acetonitrile (solvent B). A consistent flow rate of 1 mL/min was employed. The gradient elution program was performed as follows: 0–10 min: 90→10% (B); 10–20 min: 80→20% (B); 20–25 min: 75→25% (B); 25–30 min: 70→30% (B); 30–40 min: 50→50% (B); 40–50 min: 40→60% (B); 50–60 min: 97→3% (B); 60–65 min: 97→3% (B) [30].The injection volume was 10 μL.

#### 3.3.3. Methodological Validation of the HPLC Quantification Method

Linear relationship: Linear regression equations for 7 compounds were established by the peak area (Y) against the compound concentration (X). Precision: The precision of the HPLC system was evaluated using the relative standard deviation (RSD%) of six consecutive injections. Stability: The stability of the sample solution was assessed by analyzing the same solution at 0, 2, 4, 6, 8, and 10 h. Repeatability: Repeatability was determined by independently preparing six sample solutions and calculating the RSD% of the contents. Recovery: The recovery test was performed by adding known amounts of rosmarinic acid, caffeic acid, apigenin, luteolin, luteolin-7-O-glucoside, apigenin-7-O-glucoside, and 5-HMF to six portions of SFPF solution, respectively. The recovery and corresponding RSD% were then calculated.

### 3.4. Analysis of the Relationship Between Color and Chemical Composition of PF and SFPF

#### 3.4.1. Image Acquisition System

The image acquisition system consists of two main components: a smartphone and a photography studio measuring 500 × 400 × 400 mm^3^ (detailed image acquisition parameters are listed in Table S8), equipped with fill lights on both sides to ensure uniform illumination of the sample. The lights are turned on half an hour before sampling to ensure consistent lighting. The darkroom has a light-blocking rate of 99%, which minimizes interference from external light sources and ensures image accuracy. Throughout the entire photography process, the studio is completely sealed, and all images are captured manually from a fixed vertical distance of 200 mm above the darkroom [23].

#### 3.4.2. Image Processing Workflow in Python

We used PyCharm 2024.3.6.1 for code development and debugging, with Python 3.12.9 as the programming language. The image processing workflow was performed with reference to the method reported by Pan et al. [23]. Images were converted to grayscale pixels and subjected to Gaussian filtering to reduce noise for subsequent analysis. The foreground and background of the images were distinguished, followed by segmentation of pixel groups. The watershed algorithm was employed to sequentially segment each block of pixels, ensuring that each pixel corresponded to a unique region. Images were saved, and color information was extracted, including R (red), G (green), B (blue), L* (lightness), a* (green to red), and b* (blue to yellow). L* and ΔE* values were calculated using the following formulas:

$$L^{*}\;=\;0.3R\;+\;0.6G\;+\;0.1B$$

$$\Delta E^{*}=\sqrt{{L^{*}}^{2}+{a^{*}}^{2}+{b^{*}}^{2}}$$

The color data were saved in an Excel file for further analysis. This method demonstrated good stability and precision in methodological evaluations; specific details shown in Table S9.

### 3.5. Data Analysis

Origin 2025b software is used for data visualization and Design-Expert 13.0 software for RSM analysis. The peak area data of PF and SFPF were imported into SIMCA 14.1 software for PCA, HCA and OPLS-DA, using the Chinese Materia Medica chromatographic fingerprint similarity evaluation system (2012 version) software for acquired fingerprint and similarity. Statistical analysis was performed in SPSS 27.0 software. Data were expressed as mean ± SD, and independent-samples t-test was used for inter-group comparison. A *p* < 0.05 was considered statistically significant.

## 4. Discussion

In terms of processing technique, Luo et al. optimized the processing parameters of SFPF with rosmarinic acid and luteolin as evaluation indexes, and recommended the conditions of 190 °C, 4 min and 60 times/min [5]. Our research found that under the conditions of processing time 4.5 min, processing temperature 200 °C, dosage 30 g and stir-frying number 45 times/min exhibited a high comprehensive score. There are slight differences in specific parameters between studies; the overall changing trends are consistent, both confirming that moderate temperature and short-time stir-frying are the optimal processing conditions. This also verifies the rationality of the traditional TCM processing theory that “processing degree should be moderate; insufficient processing fails to guarantee efficacy, while excessive processing leads to loss of original nature and flavor” [31].

TCM fingerprint refers to the chromatogram of common characteristic peaks or characteristic signals that can represent the overall chemical characteristics of TCM. It can fully reflect the synergistic characteristics of multiple components and avoid the one-sidedness of a single index. With strong specificity, it can effectively distinguish differences in medicinal origins, producing areas, and processing techniques, providing a scientific basis for the overall quality evaluation of TCM [32].

In this study, the combination of chemical pattern recognition and fingerprinting was employed to illustrate the differences in chemical characteristics between PF and SFPF. Through comparison with reference standards, the new compound detected in SFPF was identified as 5-hydroxymethylfurfural (5-HMF), which was consistent with the findings reported by Li et al. [29]. 5-HMF is an important product of the Maillard reaction and serves as one of the chemical bases responsible for the color change in SFPF. In addition, its pharmacological activity remains controversial. Mayada et al. [33] demonstrated that long-term exposure to high concentrations of 5-HMF could induce cytotoxicity. Furthermore, it may irritate the respiratory tract, skin, and mucous membranes, and it poses certain carcinogenic risks. Research found that 5-HMF was closely associated with the regulation of renin release in the kidney, direct damage to vital organs such as the liver and kidney, and the induction of specific immune responses [34,35]. However, Yang et al. [36] reported that moderate consumption of fish containing 5-HMF could protect the human body from peroxides induced by oxidative stress in hepatocellular carcinoma cells. Zhang et al. [37] discovered that appropriate addition of 5-HMF could delay food spoilage and extend the shelf life of dairy products.

Among the differential compounds screened out, rosmarinic acid, caffeic acid and luteolin are closely associated with the therapeutic effects of Perillae Fructus. This provides a material basis for the enhanced efficacy of stir-fried Perillae Fructus in descending qi, relieving cough and calming asthma. Other studies showed that rosmarinic acid exhibits promising effects in the treatment of asthma, amelioration of acute lung injury, and antioxidant activity [8,38,39]. Caffeic acid possesses anti-tumor and lung tissue protective properties [40,41,42]. Notably, the content of luteolin was significantly elevated after stir-frying. Research reported that luteolin can alleviate pulmonary inflammation by regulating the JAK_1_/STAT_3_ signaling pathway, which makes it a crucial bioactive substance for the treatment of asthma and allergic cough [43]. The increased accumulation of luteolin may serve as an important material basis for the strengthened cough-relieving and anti-asthma effects of SFPF. Accordingly, these four differential compounds can be recommended as potential quality evaluation markers of SFPF in future studies, so as to provide a reliable reference for its quality evaluation.

In this study, for the purpose of objectively quantifying the color changes in SFPF, we determined the chromaticity values and chemical component contents of 36 batches of PF and SFPF powders, and further conducted correlation and regression analyses. Regarding the correlation between chemical components and chromaticity value variations, L* showed a positive correlation with rosmarinic acid and caffeic acid. In other words, the reduction in L* corresponded to lower contents of these two constituents. This result is consistent with the findings reported by Wang et al. [44]. They confirmed reduced rosmarinic acid and caffeic acid in SFPF, which may result from thermal destruction of related components. Yan et al. [45] found that the L* of scorched *Gardeniae Fructus* was significantly negatively correlated with 5-HMF content, indicating that elevated processing temperature promotes the Maillard reaction between carbohydrates and amino acids to generate melanoidins, resulting in a reduced L*. This study established a workflow for chromaticity value extraction from images, which only requires a smartphone and stable shooting conditions without specialized instruments, thus featuring lower cost. By establishing regression equations capable of predicting chemical component contents based on chromaticity values, this method can be used for the simple and rapid identification of SFPF quality in practical production.

## 5. Conclusions

In this study, the processing parameters of SFPF were optimized using the Box–Behnken design (BBD) coupled with response surface methodology (RSM). The results demonstrated that SFPF prepared under the conditions of processing time 4.5 min, processing temperature 200 °C, dosage 30 g and stir-frying number 45 times/min exhibited a high comprehensive score, providing an excellent material basis for the preparation of SFPF. A total of 11 common peaks were identified in 36 batches of PF and SFPF by HPLC fingerprinting, and four differential components were screened. Correlation analysis between chemical compound and color was further conducted. Data on the contents of seven compounds and colorimetric values of 36 batches of PF and SFPF were collected. The L* was negatively correlated with 5-HMF and luteolin, while the b* was positively correlated with rosmarinic acid and caffeic acid. A rapid color–compound prediction model was subsequently established.

This study systematically clarified the processing mechanism of PF. Future research may explore its in vivo biotransformation to maximize its potential value. This work not only inherits the ancient tradition of traditional Chinese medicine processing but also provides a scientific foundation for the quality control of TCM.

## Figures and Tables

**Table 1:** VIP plot of PF and SFPF.

Compound	Peak Number	VIP Value
5-HMF	1	1.8732
Caffeic acid	2	1.3385
Rosmarinic acid	8	1.1867
Luteolin	11	1.1560

**Table 2:** Regression equation, linear range, precision, stability, repeatability and recovery result of 7 compounds.

Compound	Regression Equation	Linear Range (μg/mL)	R^2^	Precision	Stability	Repeatability	Recovery	
				(RSD%, n = 6)	(RSD%, n = 6)	(RSD%, n = 6)	Mean	RSD%
Rosmarinic acid	Y = 10,092X − 146,696	60–300	0.9997	0.72	0.38	1.87	98.21	1.05
Caffeic acid	Y= 999.67X + 8.8667	5–30	1.000	0.68	0.34	1.97	98.15	1.28
Luteolin	Y = 1959.5X − 3009.7	5–50	0.9996	0.45	0.32	1.47	98.08	1.32
Apigenin	Y = 1037.8X + 2523.3	5–50	0.9992	0.51	0.41	1.67	101.95	1.45
Luteolin-7-O-glucoside	Y = 40,552X + 48,127	0.5–12	0.9997	0.48	0.42	1.17	98.12	1.25
Apigenin 7-O-glucoside	Y = 4023.6X + 5513.3	0.5–12	0.9992	0.46	0.48	0.85	99.89	1.38
5-hydroxymethylfurfural	Y = 1033.6X + 1684.4	0.5–12	0.9992	0.79	0.79	0.89	102.05	1.4

## Data Availability

Data are contained within the article and Supplementary Materials.

## Supplementary Materials

The following supporting information can be downloaded at: https://www.mdpi.com/article/10.3390/molecules31111907/s1. Table S1: The information on collected PF samples from different sources. Table S2: The results of the 29 experiments of BBD model. Table S3: The ANOVA results of the experiments of BBD model. Table S4. Experimental validation of processing techniques. Table S5: The precision, repeatability and stability evaluation of HPLC fingerprint method. Table S6: The similarity analysis of HPLC fingerprint of PF and SFPF. Table S7: Content determination of differential compounds of PF and SFPF. Table S8: Image acquisition parameters. Table S9: Methodological validation of image acquisition system. Figure S1: PF and SFPF powder surface appearance.

## Abbreviations

The following abbreviations are used in this manuscript:

PF	Perillae Fructus
SFPF	Stir-fried Perillae Fructus
HCA	Hierarchical cluster analysis
OPLS-DA	Orthogonal partial least squares discriminant analysis
PCA	Principal component analysis
TCM	Traditional Chinese medicine
