# Supplementary material for: Chemical Pattern Recognition and Color–Chromaticity Correlation Analysis for Quality Control of Stir-Fried Perillae Fructus
Source: Molecules. 2026 Jun 2;31(11):1907. doi: 10.3390/molecules31111907 (PMC13258405; doi:10.3390/molecules31111907)
Supplement: Supplementary file 1 [file molecules-31-01907-s001.zip › molecules-4307346-supplementary.pdf]

Supplementary Data

Chemical Pattern Recognition and Color-Chromaticity Correla-tion Analysis for Quality Control of Stir-Fried Perillae Fructus

Liangying Li [1], Xiaobin Deng [1], Pengbo Wang [1], Nina, Zeng [1], Jing Hu [1,2,*]

[1] School of Chinese Materia Medica, Tianjin University of Traditional Chinese Medicine, Tianjin 301617, China

[2] Tianjin Key Laboratory of Therapeutic Substance of Traditional Chinese Medicine, Tianjin 301617, China

[*] Correspondence：hutcm8181@tjutcm.edu.cn

Number	Content	Page
1	**Table S1:** The information on collected PFs samples from different sources	2
2	**Table S2:** The results of the 29 experiments of BBD model	3
3	**Table S3**: The ANOVA results of the experiments of BBD model	4
4	**Table S4:** Experimental validation of processing techniques	4
5	**Table S5:** The precision, repeatability and stability evaluation of HPLC fingerprint method of PF	5
6	**Table S6:** Results of similarity evaluation of 36 batches of PF and SFPF	6
7	**Table S7:** 7 compounds content determination of differential compounds of PF and SFPF	7
7	**Table S8:** Image acquisition parameters.	10
8	**Table. S9** Methodologically validation of image acquisition system.	10
9	**Figure. S1**：PF and SFPF surface appearance	11

Table S1 Information on the origin of PF

NO.	Herbal medicine batch number	Origin	NO.	Herbal medicine batch number	Origin
1	2403002	Hebei, China	19	20250615	Heilongjiang, China
2	2412001	Hebei, China	20	20250201	Anhui, China
3	C245250301	Hebei, China	21	20240701	Anhui, China
4	20250511	Hebei, China	22	20241110	Anhui, China
5	20250605	Hebei, China	23	20250505	Anhui, China
6	20250609	Shanxi, China	24	20250601	Anhui, China
7	20250515	Shanxi, China	25	20250605	Anhui, China
8	20250609	Shanxi, China	26	20250610	Jiangsu, China
9	A250218	Shanxi, China	27	20250612	Jiangsu, China
10	A250210	Shanxi, China	28	20250212	Jiangsu, China
11	250303501	Shanxi, China	29	20250601	Guangxi, China
12	202410152	Shanxi, China	30	250301	Guangxi, China
13	A250101	Shanxi, China	31	250101	Sichuang, China
14	240901	Shanxi, China	32	240725-1	Guangdong, China
15	20230305	Shanxi, China	33	241101	Hubei, China
16	20240319	Heilongjiang, China	34	20241209	Jiangxi, China
17	241001	Heilongjiang, China	35	GT240106073	Gansu, China
18	230301	Heilongjiang, China	36	240501	Henan, China

Table S2 The results of the 29 experiments of BBD model

No.	Processing time(min) A	Processing temperature (℃) B	Dosage(g) C	Sir-frying number (times/min) D	Comprehensive score
1	4	200	50	60	73.44
2	4	240	40	60	65.2
3	4	200	40	50	75.7
4	5	200	50	50	68.96
5	4	200	30	40	71.45
6	4	240	30	50	66.56
7	4	160	50	50	59.4
8	5	240	40	50	62.82
9	3	200	30	50	67.47
10	3	200	40	40	67.11
11	5	200	30	50	74.7
12	4	160	30	50	60.25
13	4	200	40	50	69.13
14	4	240	40	40	62.41
15	3	200	40	60	67.01
16	4	200	40	50	66.31
17	4	200	50	40	74.27
18	4	200	40	50	71.85
19	4	200	30	60	70.76
20	5	200	40	60	69.16
21	4	160	40	60	51.24
22	3	160	40	50	49.08
23	4	240	50	50	63.89
24	3	200	50	50	73.29
25	3	240	40	50	61.32
26	5	200	40	40	75.77
27	4	200	40	50	70.32
28	4	160	40	40	57.91
29	5	160	40	50	60.55

Table S3 The ANOVA results of the experiments of BBD model

Source	Sum of Squares	df	Mean Square	F-value	*p*-value	significant
Model	1204.64	14	86.05	21.94	< 0.0001	**
A	59.32	1	59.32	15.12	0.0030	**
B	159.65	1	159.65	40.70	< 0.0001	**
C	0.3536	1	0.3536	0.0902	0.7701	
D	12.22	1	12.22	3.12	0.1080	
AB	24.85	1	24.85	6.34	0.0305	*
AC	33.41	1	33.41	8.52	0.0153	*
AD	10.60	1	10.60	2.70	0.1313	
BC	0.8281	1	0.8281	0.2111	0.6557	
BD	22.37	1	22.37	5.70	0.0381	*
CD	0.0049	1	0.0049	0.0012	0.9725	
A^2	40.33	1	40.33	10.28	0.0094	**
B^2	503.62	1	503.62	128.39	< 0.0001	**
C^2	0.4059	1	0.4059	0.1035	0.7543	
D^2	20.86	1	20.86	5.32	0.0438	*
R^2	0.9685					
Radj2	0.9243					
Residual	39.23	10	3.92			
Lack of fit	0.30243	3	0.1008	3.72	0.1184	
Pure error	0.1084 4	4	0.0271			
Cor Total	1243.87	24				

Table S4 Experimental validation of processing techniques

	TF mg/g	TPA mg/g	TP %	PFO %	Comprehensive score
1	15.50	9.20	9.27	35.67	77.46
2	16.64	10.16	9.41	36.63	78.19
3	15.48	10.29	9.67	37.32	81.65
Average	3.54	10.21	9.45	36.54	79.09
RSD%	0.08	0.06	0.20	1.82	0.39

Table S5 The precision, repeatability and stability evaluation of HPLC fingerprint method of PF.

Common peaks	Precision (RSD%, n = 6)		Repeatability (RSD%, n = 6)		Stability (RSD%, n = 6)	
	Relative Peak area	Relative Retention time	Relative Peak area	Relative Retention time	Relative Peak area	Relative Retention time
1	2.77	0.09	1.52	0.20	2.76	0.07
2	1.14	0.18	1.66	0.16	2.94	0.08
3	0.77	0.06	0.47	0.06	1.74	0.09
4	1.52	0.07	0.83	0.08	2.58	0.11
5	1.22	0.04	0.70	0.07	2.93	0.09
6	3.72	0.04	3.16	0.07	1.89	0.06
7	0.00	0.00	0.00	0.00	0.00	0.00
8	0.62	0.04	0.15	0.04	1.45	0.02
9	1.23	0.005	0.17	0.006	1.26	0.004
10	0.84	0.04	2.39	0.03	2.78	0.03
11	0.59	0.04	1.08	0.04	2.18	0.02

Table S6 Results of similarity evaluation of 36 batches of PF and SFPF

		PF	
Number	Similarity	Number	Similarity
S1	0.936	S19	0.988
S2	0.985	S20	0.973
S3	0.955	S21	0.967
S4	0.997	S22	0.961
S5	0.986	S23	0.994
S6	0.994	S24	0.938
S7	0.977	S25	0.918
S8	0.957	S26	0.994
S9	0.996	S27	0.996
S10	0.956	S28	0.914
S11	0.937	S29	0.993
S12	0.995	S30	0.968
S13	0.995	S31	0.988
S14	0.996	S32	0.913
S15	0.984	S33	0.977
S16	0.978	S34	0.994
S17	0.987	S35	0.996
S18	0.991	S36	0.987

Results of similarity evaluation of 36 batches of SFPF

		SFPF	
Number	Similarity	Number	Similarity
S37	0.994	S55	0.994
S38	0.989	S56	0.979
S39	0.979	S57	0.987
S40	0.996	S58	0.977
S41	0.987	S59	0.967
S42	0.996	S60	0.993
S43	0.994	S61	0.947
S44	0.992	S62	0.914
S45	0.989	S63	0.994
S46	0.997	S64	0.807
S47	0.942	S65	0.992
S48	0.949	S66	0.997
S49	0.994	S67	0.989
S50	0.993	S68	0.988
S51	0.995	S69	0.98
S52	0.939	S70	0.997
S53	0.997	S71	0.956
S54	0.97	S72	0.991

Table S7 7 compounds content determination of differential compounds of PF and SFPF

	NO	Type	Rosmarinic acid	Caffeic acid	Luteolin	Luteolin-7-O-glucoside	Apigenin	Apigenin 7-O-glucoside	5-HMF
1	2403002	PF	3.01±0.05	0.18±0.03	0.23±0.12	0.06±0.05	0.14±0.02	0.04±0.05	0
		SFPF	2.91±0.03	0.17±0.01	0.24±0.03	0.05±0.06	0.17±0.01	0.04±0.02	0.06±0.02
2	2412001	PF	3.04±0.02	0.18±0.10	0.22±0.04	0.06±0.03	0.14±0.03	0.05±0.02	0
		SFPF	2.92±0.04	0.17±0.20	0.23±0.10	0.05±0.05	0.16±0.04	0.04±0.01	0.06±0.01
3	C245250301	PF	3.00±0.05	0.18±0.03	0.23±0.12	0.05±0.07	0.15±0.02	0.05±0.06	0
		SFPF	2.90±0.06	0.17±0.02	0.24±0.03	0.05±0.09	0.17±0.04	0.04±0.05	0.05±0.02
4	20250511	PF	3.04±0.03	0.18±0.02	0.23±0.04	0.06±0.10	0.14±0.05	0.04±0.02	0
		SFPF	2.92±0.01	0.17±0.03	0.24±0.03	0.05±0.12	0.16±0.09	0.04±0.03	0.05±0.01
5	20250605	PF	2.99±0.02	0.17±0.04	0.23±0.03	0.06±0.11	0.15±0.07	0.05±0.02	0
		SFPF	2.89±0.03	0.16±0.05	0.24±0.03	0.05±0.04	0.17±0.08	0.04±0.05	0.04±0.05
6	20250609	PF	3.02±0.03	0.18±0.10	0.22±0.04	0.05±0.05	0.14±0.06	0.05±0.05	0
		SFPF	2.91±0.02	0.16±0.03	0.24±0.12	0.05±0.03	0.17±0.04	0.04±0.02	0.04±0.05
7	20250515	PF	3.02±0.04	0.18±0.04	0.23±0.03	0.06±0.02	0.14±0.03	0.04±0.01	0
		SFPF	2.90±0.05	0.17±0.10	0.24±0.03	0.05±0.04	0.16±0.03	0.04±0.05	0.03±0.03
8	20250609	PF	3.03±0.02	0.18±0.10	0.22±0.04	0.06±0.06	0.15±0.05	0.05±0.03	0
		SFPF	2.92±0.02	0.17±0.03	0.24±0.10	0.05±0.09	0.17±0.02	0.04±0.06	0.03±0.02
9	A250218	PF	2.98±0.04	0.17±0.04	0.23±0.11	0.05±0.02	0.14±0.03	0.05±0.02	0
		SFPF	2.89±0.03	0.16±0.03	0.24±0.02	0.05±0.14	0.16±0.02	0.04±0.06	0.03±
10	A250210	PF	3.02±0.04	0.18±0.04	0.23±0.03	0.06±0.012	0.14±0.05	0.05±0.03	0
		SFPF	2.91±0.03	0.17±0.03	0.24±0.04	0.05±0.09	0.17±0.01	0.04±0.03	0.03±0.02
11	250303501	PF	3.00±0.03	0.17±0.03	0.23±0.09	0.06±0.07	0.15±0.05	0.05±0.05	0
		SFPF	2.90±0.04	0.16±0.04	0.24±0.08	0.05±0.06	0.16±0.03	0.04±0.09	0.02±0.12
12	202410152	PF	3.03±0.02	0.18±0.05	0.23±0.07	0.05±0.06	0.14±0.03	0.05±0.03	0

		SFPF	2.92±0.04	0.17±0.05	0.24±0.01	0.06±0.03	0.17±0.05	0.04±0.03	0.02±0.01
13	A250101	PF	2.99±0.03	0.17±0.04	0.23±0.05	0.06±0.02	0.14±0.03	0.04±0.05	0
		SFPF	2.88±0.05	0.16±0.05	0.24±0.02	0.05±0.05	0.16±0.09	0.04±0.09	0.02±0.04
14	240901	PF	3.02±0.06	0.17±0.02	0.22±0.05	0.06±0.03	0.14±0.01	0.05±0.01	0
		SFPF	2.91±0.02	0.16±0.01	0.24±0.01	0.05±0.04	0.17±0.11	0.04±0.05	0.01±0.02
15	20230305	PF	3.02±0.03	0.18±0.03	0.22±0.03	0.06±0.04	0.15±0.05	0.05±0.02	0
		SFPF	2.90±0.03	0.16±0.03	0.24±0.01	0.05±0.01	0.17±0.01	0.04±0.01	0.01±0.04
16	20240319	PF	3.00±0.02	0.17±0.04	0.23±0.01	0.06±0.06	0.14±0.05	0.05±0.04	0
		SFPF	2.92±0.02	0.17±0.04	0.23±0.02	0.05±0.05	0.16±0.01	0.04±0.13	0.01±0.03
17	241001	PF	3.03±0.03	0.18±0.05	0.22±0.02	0.05±0.09	0.14±0.05	0.05±0.05	0
		SFPF	2.90±0.03	0.16±0.04	0.24±0.03	0.05±0.03	0.17±0.02	0.04±0.01	0.06±0.02
18	230301	PF	2.99±0.03	0.17±0.05	0.23±0.01	0.06±0.01	0.15±0.01	0.04±0.04	0
		SFPF	2.92±0.12	0.17±0.04	0.24±0.05	0.05±0.05	0.16±0.02	0.04±0.09	0.05±0.05
19	20250615	PF	3.01±0.04	0.18±0.05	0.22±0.04	0.06±0.01	0.14±0.01	0.05±0.01	0
		SFPF	2.89±0.03	0.16±0.06	0.24±0.01	0.05±0.03	0.17±0.02	0.04±0.01	0.05±0.03
20	20250201	PF	3.02±0.03	0.18±0.06	0.22±0.03	0.05±0.05	0.14±0.01	0.05±0.09	0
		SFPF	2.91±0.04	0.16±0.02	0.24±0.06	0.05±0.01	0.17±0.02	0.04±0.05	0.04±0.09
21	20240701	PF	3.01±0.04	0.17±0.01	0.23±0.03	0.06±0.02	0.15±0.03	0.04±0.09	0
		SFPF	2.88±0.04	0.16±0.02	0.23±0.04	0.06±0.02	0.16±0.01	0.04±0.03	0.04±0.01
22	20241110	PF	3.03±0.03	0.18±0.05	0.22±0.03	0.06±0.05	0.14±0.02	0.05±0.01	0
		SFPF	2.90±0.02	0.16±0.03	0.24±0.05	0.06±0.03	0.17±0.05	0.04±0.02	0.04±0.02
23	20250505	PF	3.01±0.03	0.17±0.05	0.23±0.09	0.06±0.02	0.14±0.01	0.05±0.04	0
		SFPF	2.91±0.05	0.17±0.12	0.24±0.05	0.05±0.04	0.16±0.05	0.04±0.05	0.03±0.03
24	20250601	PF	2.98±0.03	0.17±0.04	0.23±0.06	0.06±0.02	0.14±0.01	0.04±0.01	0
		SFPF	2.89±0.03	0.16±0.02	0.23±0.01	0.05±0.02	0.17±0.02	0.04±0.09	0.03±0.02
25	20250605	PF	3.02±0.04	0.18±0.03	0.22±0.02	0.05±0.01	0.15±0.01	0.05±0.01	0

		SFPF	2.91±0.05	0.16±0.03	0.24±0.01	0.05±0.03	0.17±0.02	0.04±0.01	0.03±0.02
26	20250610	PF	3.00±0.03	0.17±0.02	0.22±0.03	0.06±0.10	0.14±0.02	0.04±0.02	0
		SFPF	2.90±0.04	0.16±0.01	0.24±0.02	0.05±0.11	0.16±0.01	0.04±0.04	0.02±0.02
27	20250612	PF	3.03±0.02	0.18±0.05	0.23±0.01	0.06±0.012	0.14±0.02	0.05±0.01	0
		SFPF	2.92±0.03	0.17±0.02	0.24±0.03	0.05±0.02	0.16±0.03	0.04±0.05	0.02±0.01
28	20250212	PF	2.99±0.03	0.17±0.02	0.22±0.03	0.05±0.03	0.15±0.06	0.05±0.01	0
		SFPF	2.88±0.02	0.16±0.05	0.24±0.07	0.05±0.02	0.17±0.04	0.04±0.05	0.02±0.01
29	20250601	PF	3.01±0.04	0.17±0.02	0.23±0.05	0.06±0.01	0.14±0.02	0.04±0.02	0
		SFPF	2.90±0.02	0.16±0.01	0.23±0.02	0.05±0.05	0.16±0.01	0.04±0.04	0.02±0.02
30	250301	PF	3.02±0.03	0.18±0.03	0.22±0.02	0.05±0.01	0.14±0.04	0.05±0.01	0
		SFPF	2.91±0.03	0.16±0.01	0.24±0.03	0.05±0.04	0.17±0.05	0.04±0.01	0.02±0.03
31	250101	PF	3.00±0.03	0.17±0.05	0.23±0.04	0.06±0.01	0.14±0.06	0.05±0.04	0
		SFPF	2.89±0.06	0.16±0.01	0.24±0.05	0.05±0.02	0.17±0.06	0.04±0.05	0.02±0.06
32	240725-1	PF	3.03±0.05	0.18±0.03	0.22±0.05	0.06±0.03	0.15±0.01	0.04±0.01	0
		SFPF	2.92±0.05	0.17±0.02	0.24±0.01	0.05±0.04	0.16±0.05	0.04±0.01	0.01±0.02
33	241101	PF	2.98±0.04	0.17±0.02	0.23±0.04	0.05±0.02	0.14±0.01	0.05±0.03	0
		SFPF	2.90±0.03	0.16±0.05	0.24±0.01	0.05±0.04	0.17±0.05	0.04±0.01	0.06±0.06
34	20241209	PF	3.01±0.02	0.17±0.03	0.22±0.01	0.06±0.03	0.14±0.02	0.05±0.01	0
		SFPF	2.91±0.02	0.16±0.04	0.23±0.10	0.05±0.05	0.16±0.08	0.04±0.01	0.05±0.02
35	GT240106073	PF	3.02±0.03	0.17±0.05	0.22±0.05	0.06±0.02	0.15±0.04	0.04±0.05	0
		SFPF	2.89±0.04	0.16±0.05	0.24±0.13	0.05±0.02	0.17±0.08	0.05±0.01	0.05±0.01
36	240501	PF	3.00±0.06	0.17±0.07	0.23±0.01	0.05±0.07	0.14±0.07	0.05±0.04	0
		SFPF	2.90±0.04	0.16±0.04	0.24±0.01	0.05±0.09	0.16±0.06	0.04±0.01	0.04±0.01

Table S8 Image acquisition parameters

Indicator	Parameters
Device model	Apple iPhone 16
Camera height	50cm
Rear camera resolutio	48MP
Light source height	40cm
Illumination intensity	Lux
Image size	3024×4032
Aperture size	F/1.6
Exposure time	1/121s

Table S9 Methodologically validation of image acquisition system

Color parameter	Precision (RSD%, n = 6)	Repeatability (RSD%, n = 6)	Stability (RSD%, n = 6)
*L**	2.77	1.52	2.76
*a**	1.14	1.66	2.94
*b**	0.77	0.47	1.74

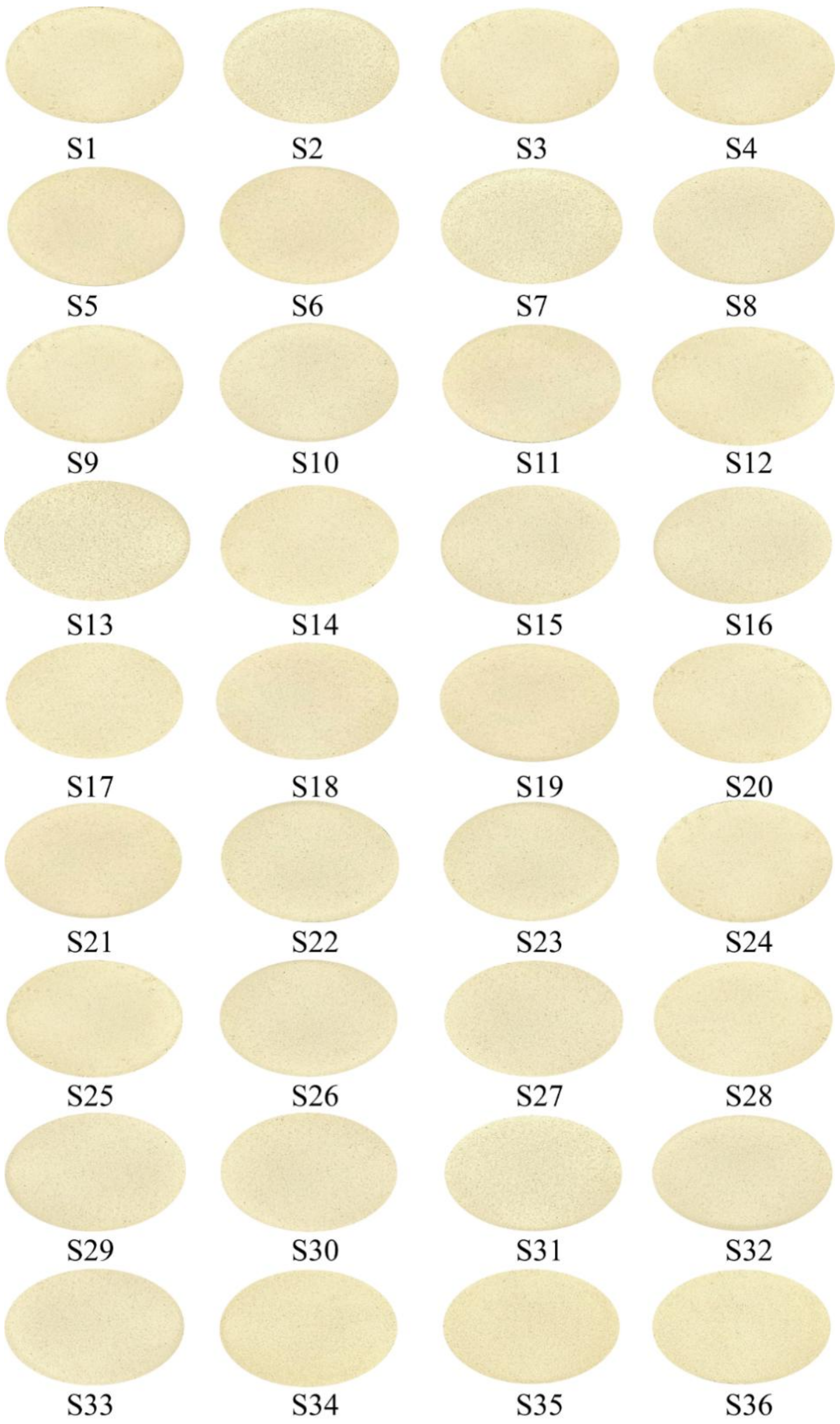
S1
S2
S3
S4
S5
S6
S7
S8
S9
S10
S11
S12
S13
S14
S15
S16
S17
S18
S19
S20
S21
S22
S23
S24
S25
S26
S27
S28
S29
S30
S31
S32
S33
S34
S35
S36

PF

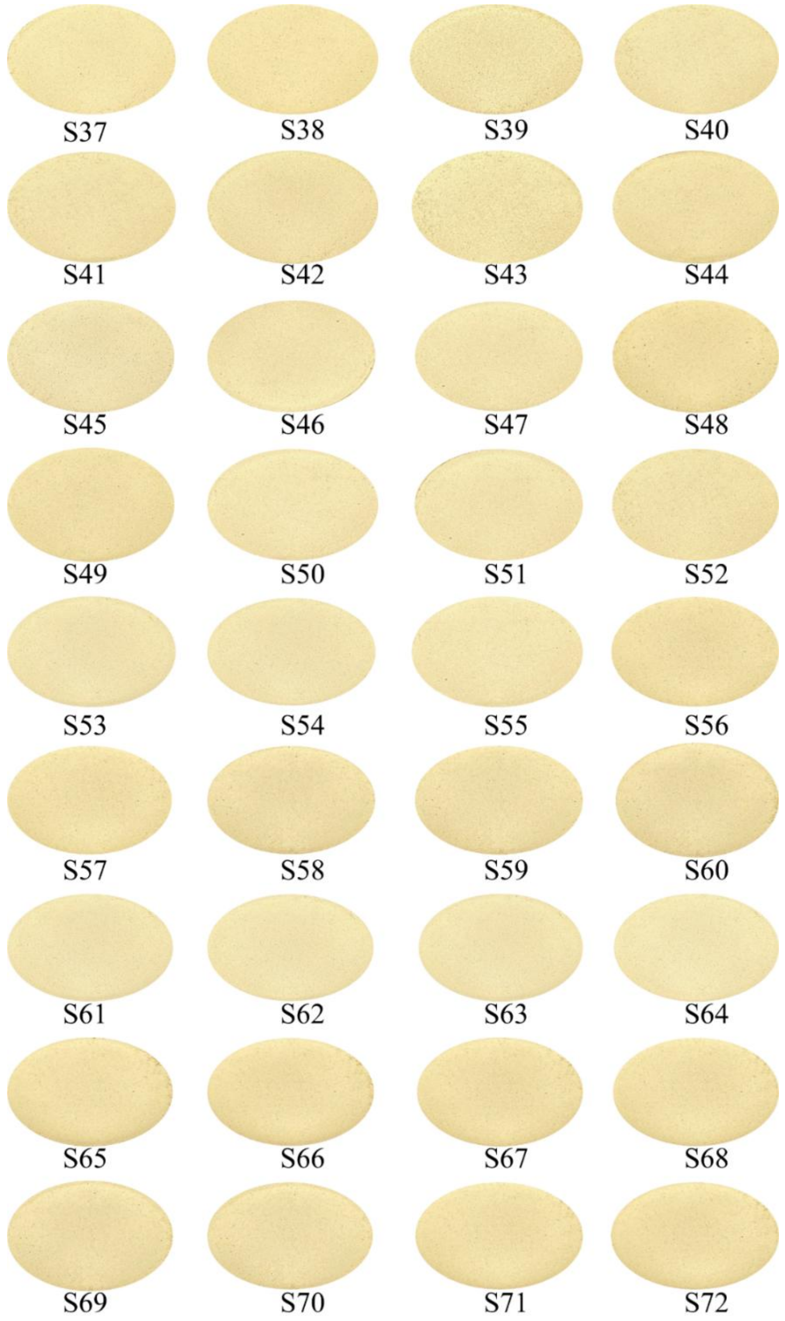

Figure S1 PF and SFPF powder surface appearance
